# Employee innovation during office work, work from home and hybrid work

**DOI:** 10.1038/s41598-024-67122-6

**Published:** 2024-07-24

**Authors:** Michael Gibbs, Friederike Mengel, Christoph Siemroth

**Affiliations:** 1 University of Chicago, Chicago, USA; 2 Institute of Labor Economics, Bonn, Germany; 3 University of Essex, Colchester, UK; 4 University of Heidelberg, Heidelberg, Germany

**Keywords:** Collaboration, Coordination, Innovation, Working from home, Hybrid work, Telecommuting, Human behaviour, Psychology and behaviour

## Abstract

The Covid-19 pandemic forced firms globally to shift workforces to working from home [WFH]. Firms are now struggling to implement a return to working from the office [WFO], as employees enjoy the significant benefits of WFH for their work-life balance. Therefore many firms are adopting a hybrid model in which employees work partly from the office and partly from home. We use unique and detailed data from an Indian IT services firm which contains a precise measure of innovation activity of over 48,000 employees in these three work environments. Our key outcomes are the quantity and quality of ideas submitted by employees. Based on an event study design, the quantity of ideas did not change during the WFH period as compared to WFO, but the quality of ideas suffered. During the later hybrid period, the quantity of submitted ideas fell. In the hybrid phase innovation suffered particularly in teams which were not well coordinated in terms of when they worked at the office or from home. Our findings suggest that remote and hybrid work modes may inhibit collaboration and innovation.

## Introduction

During the Covid-19 pandemic, an enormous shift towards working from home [WFH] occurred across the globe. As the pandemic has waned, many employees hope to maintain use of WFH, because of the significant work-life balance benefits, including more flexibility in work time, lower commute time, and in many cases the opportunity to live somewhere that is more desirable. However, most employers are now adopting hybrid work modes in which employees must work at the office [WFO] some of the time, but are allowed to work from home at other times. Little is yet known about the effects of hybrid work in particular^1–3^.

CEOs of many firms are worried that remote work modes cause losses in intangibles that benefit from in-person interactions among employees; e.g., on-boarding and development of new employees, nurturing corporate culture, and fostering collaboration. One particular concern that is frequently expressed by corporate leaders is that WFH and hybrid work modes harm innovation. For example – and somewhat ironically – the CEO of Zoom, the leading online collaboration application, recently mandated that employees work more from the office, specifically citing a decline in innovation^4^.

In this paper, we provide the first empirical evidence on this important question. Innovation is notoriously hard to study^5,6^. It is typically measured by looking at patents or trademarks^7^, but that says little about how innovation takes place within the firm. Because of the difficulty of obtaining good measures of innovation within the firm, very little is known even descriptively about how working from home or hybrid work modes impact innovation.

Using an event study design, we analyze unique and high quality measures of innovation activity for over 48,000 employees, in WFO, WFH and hybrid work modes. The subjects of our study are highly educated IT professionals – virtually all have college or advanced degrees in an engineering field. Their work involves significant cognitive tasks as well as collaboration. Innovation is not a core part of their work, but the company strongly encourages innovation and pays monetary incentives to foster innovation at work.

We find that innovation suffers during remote work. During the WFH period, employees suggested ideas at the same rate as during the initial WFO period, but those ideas had lower average quality. By contrast, during the ensuing hybrid period, the rate with which new ideas were generated fell.

Why does innovation decrease in remote or hybrid work? Our evidence suggests that hybrid work raises the cost of collaboration. Innovation often occurs through random, spontaneous “watercooler” interactions between employees. Such “productive accidents” are less likely to occur when all employees work from home, requiring firms to provide substitute channels where innovation can happen (e.g., “virtual coffee rooms”). One question is whether virtual communication is equally conducive to the generation of new ideas as face to face communication^8^. In hybrid mode, an additional coordination problem arises if some employees are in “virtual coffee rooms” while others are in actual coffee rooms^9^. By analyzing office swipe-in data from the hybrid work phase, we find that innovation drops particularly sharply in teams with high variation in office presence compared to teams that are less scattered.

These findings are significant, as they suggest that concerns about losses in innovation are valid. Moreover, our evidence indicates that a hybrid work mode may also exhibit some of these problems, so that firms will have to find ways to mitigate these downsides if they want to offer more opportunities for employees to work partly from home.

## Methods

For this study we used data provided by HCL Technologies, one of the world’s largest IT services companies, with headquarters in India. All necessary consent has been obtained by the company in line with current regulations. As we use only anonymous archival data ethical approval was not required. The study was carried out in accordance with relevant guidelines and regulations. Additional details about the company are in Supplement A. See^10^ and^11^ for related analyses of HCL data, on employee innovation, and productivity while working from home during the pandemic. The company had three work modes during the sample period. In the first phase, employees worked from the office. When the Covid-19 pandemic hit, the company abruptly switched to working from home. When the pandemic abated, the company moved to a hybrid work scheme in which employees were allowed to work partially from home, but were also expected to work regularly from the office. Thus, all three work modes were company-wide policy, so employees were not able to switch from one work mode to the other. The company provided us with data on employee characteristics, and information on all innovation ideas submitted to the employee suggestion system.

IT services is a highly competitive industry. For many years, HCL has pursued a strategy emphasizing innovation for its clients, with the goals of being more differentiated from competitors, and more like long-term partners for clients^12^. As part of this effort, the company has taken significant steps to instill a culture in which all employees see innovation as a key part of their job. A cornerstone of this is the Idea Portal. This is an Intranet system which all employees can use to submit new ideas, small or large, that may benefit the company or its clients. All employees are encouraged to participate in the Idea Portal. This system is viewed by top executives, including the CEO, as highly valuable for HCL. Supervisors and executives have always had strong implicit incentives to evaluate ideas carefully and seriously.

**Figure 1:**
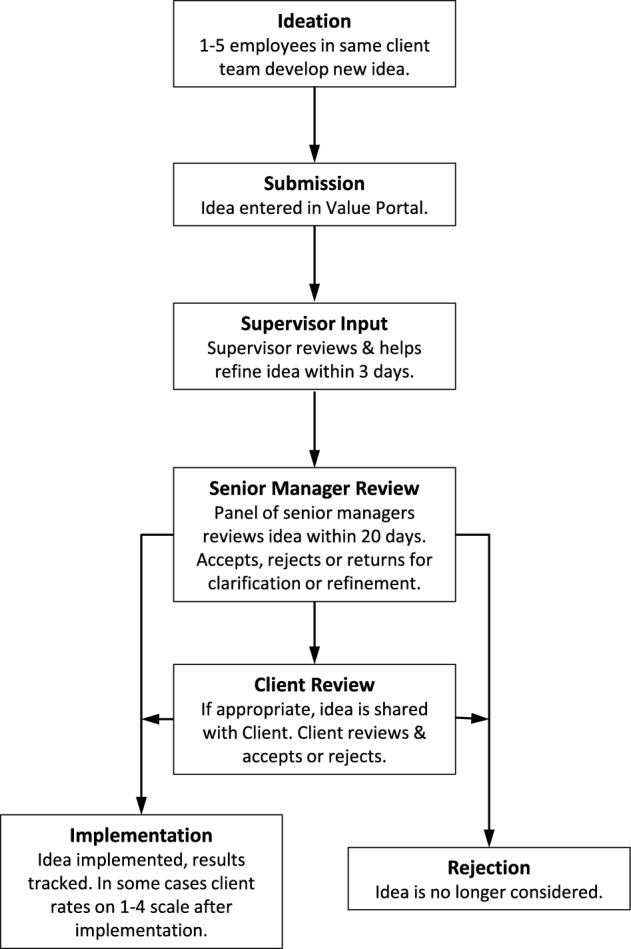
Process for Evaluating New Ideas.

Figure 1 illustrates the process by which ideas are evaluated. Briefly, employees may come up with new ideas spontaneously, or they may try to ideate formally to generate new ideas. This may be done individually or with colleagues. If the employee has a new idea that he or she believes is valuable, they can (with up to 4 colleagues) submit a description of the idea, including estimates of resources needed to implement, and potential benefits, on the Idea Portal. Within three days, the supervisor is expected to review the idea, and either reject it, help the employee refine it, or approve it for consideration. If an idea is approved for consideration, it is reviewed within three weeks by a panel of executives, who can either reject or approve the idea. If approved and likely to have direct effect on a client, the idea may then be submitted to the client for final approval. Accepted ideas are then implemented. For a more detailed description of the process, see^10^. It is important to note that this process did not change during our study period, specifically also not during the WFH and hybrid periods. Since the system was developed many years ago, it was robust well before the start of our sample period. The system was implemented on HCL’s Intranet and ideas were processed and evaluated online even during the WFO period.

We aggregate the number of ideas that each employee submitted in every month, and match these to recreated employee rosters (see Supplement A). Employees that are not found in the Idea Portal data in a given month have not submitted ideas and are therefore counted as zeros. Our key outcomes are the Quantity and Quality of ideas that employees suggest; see Supplement B.1 for details.

To quantify the WFH and hybrid work effects, and to control for employee and team time-invariant variables (via employee and team fixed effects), we use the following regression analyses, which are an event study with controls. Our main specification exploits differences in outcomes for each employee, when working from home and working in a hybrid mode compared to working in the office, controlling for employee and customer team fixed effects. The unit of observation is the employee-month. Index the employee by *i* and the month by $t=1,2,\ldots$. For outcome variable $y_{it}$, we estimate by OLS:

1 $$\begin{aligned} \begin{aligned} y_{it}=\alpha _i+\beta _1 \text {WFH}_t+\beta _2 \text {Hybrid}_t+\sum _j\gamma _j \text {CustomerTeam}_{jit}+\sum _{s}\delta _s \text {Month}_{st}+\zeta t+\varepsilon _{it}, \end{aligned} \end{aligned}$$

where $\alpha _i$ is the employee fixed effect, WFH is a dummy variable indicating months working from home, Hybrid is a dummy variable indicating months in a hybrid mode (employees individually choose where to work), and CustomerTeam$_{jit}$ is a dummy variable equal to one if and only if employee *i* in month *t* was part of team *j*. Month$_{st}$ is a month (not month-year) dummy variable, so that Month$_{1t}=1$ if and only if *t* is January, Month$_{2t}=1$ if and only if *t* is February, etc. $\zeta$ is the coefficient of the linear monthly time trend to account for possible long term trends. Hence, our time controls are very flexible by controlling for both seasonal effects and a long term trend. We exclude March 2020 from regressions because our main outcome variables are aggregated to the monthly level, and working from home started in mid-March 2020. Thus, this month is neither purely WFO nor WFH. Moreover, it is likely that WFH increased in the days prior to the official WFH start, so the switch date was not clear-cut.

Informally, the estimates give us average differences in outcomes between working from home and office modes, and between hybrid and office modes, for the same employee, controlling for team effects (since employees sometimes switch teams) as well as seasonal and linear time trend controls.

## Results

### Idea quantity

**Figure 2:**
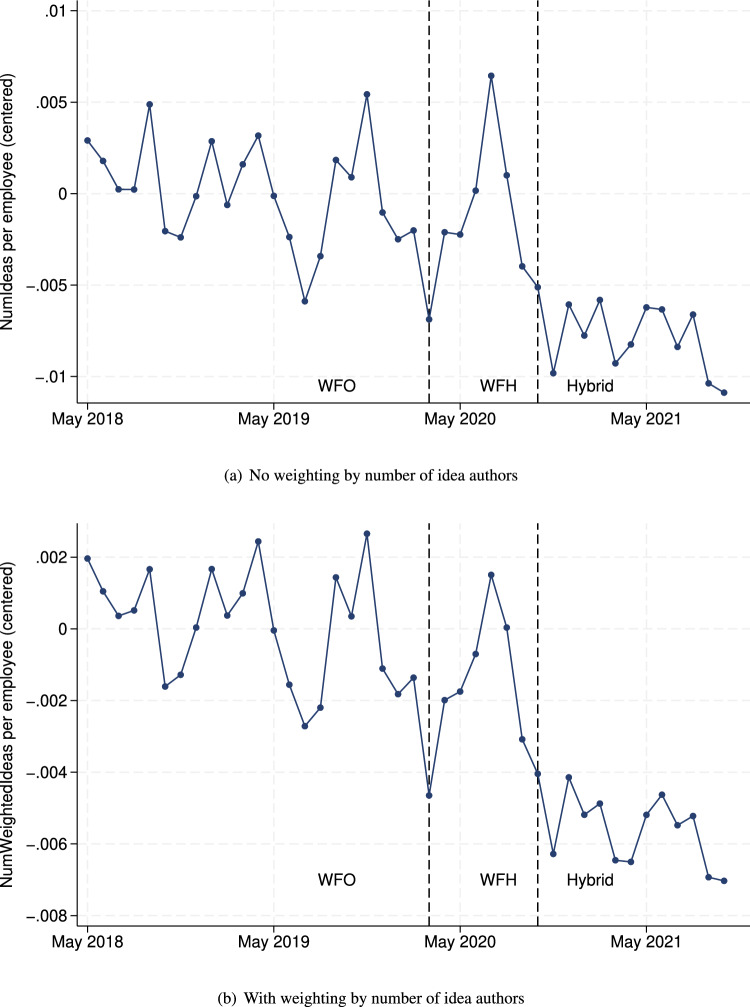
Average number of ideas submitted per employee per month after removing the linear and seasonal time trends (y-axis normalized: 0 is WFO mean). The vertical bars indicate changes in the work mode (WFO, WFH, hybrid).

The number of ideas that employees submitted in the Idea Portal was lower with hybrid work. This can be seen in Fig. 2, which plots the number of ideas per employee per month over our sample period after removing time trends. Figure 2a plots the unweighted number (the number of ideas an employee submitted, possibly with other employees as coauthors, in a given month). Figure 2b plots the weighted number of ideas (the number of ideas, each divided by the number of authors of that idea, that an employee submitted in a given month).

The precise estimates of Eq. 1 can be found in Table 1, which reports WFH and hybrid work effect estimates relative to WFO, the base category. WFH did not significantly change the number of ideas submitted by an employee in a constant time window, relative to work from the office (Table 1). This is true for all four idea quantity measures we use: (i) NumIdeas, the number of ideas an employee submitted (possibly with other employees as coauthors) in a given month; (ii) NumWeightedIdeas, the number of ideas, each divided by the number of authors of that idea, that an employee submitted in a given month; (iii) NumIdeasMS3, a three month moving sum of NumIdeas for each employee; (iv) NumWeightedIdeasMS3, a three month moving sum of NumWeightedIdeas with (iii) and (iv) only defined if all three months are part of the same work mode. Table B.1 in Supplement B provides summary statistics for these variables. Note also that focusing on the specification in column (1) of Table 1 we have more than $95\%$ power to detect an effect size equal to the Hybrid effect or an effect size equal to half of the Hybrid effect at the 5 percent level.

Hence, when it comes to the quantity of ideas, the often-expressed worry that WFH hampers innovation does not find support in our data. However, during hybrid work there was a significant reduction in the number of ideas, relative to both WFO and WFH. Again, this is true for all four idea quantity measures and hence a robust finding. The magnitude of the change in idea quantity during hybrid work is meaningful, since the base rate is small. According to summary Table B.1, employees generated 0.009 ideas per month during WFO on average. This means it takes an employee about 111 months, or a bit more than 9 years, to generate an idea. During hybrid work, employees generated 0.007 ideas per month on average, which corresponds to 143 months or just below 12 years for one idea. These numbers correspond to a drop of 22% of ideas per employee per month in the switch from WFO to hybrid work.

**Table 1:** OLS: Average WFH and hybrid work effects.

Dependent variable	(1)	(2)	(3)	(4)
	NumIdeas	NumWeightedIdeas	NumIdeasMS3	NumWeightedIdeasMS3
WFH	0.001	−0.000	0.005	−0.000
	(0.001)	(0.000)	(0.004)	(0.002)
Hybrid	−0.006***	−0.004***	−0.024***	−0.016***
	(0.001)	(0.001)	(0.003)	(0.002)
Employee FE	Yes	Yes	Yes	Yes
Team FE	Yes	Yes	Yes	Yes
Month FE	Yes	Yes	Yes	Yes
Linear time trend	Yes	Yes	Yes	Yes
Test $\text {WFH}=\text {Hybrid}$ (*p*-value)	0.000	0.000	0.000	0.000
WFO Mean	0.009	0.006	0.030	0.018
Observations	1060648	1060648	867234	867234
Clusters	48110	48110	44453	44453

*Note:* NumIdeas and NumWeightedIdeas are the number of submitted ideas and the number of submitted ideas inversely weighted by the number of idea authors, respectively. NumIdeasMS3 and NumWeightedIdeasMS3 are moving 3-month sums of the number of submitted ideas and the number of submitted ideas inversely weighted by the number of idea authors, respectively. The unit of observation is the employee-month. Standard errors are shown in brackets below the point estimates, and are clustered on employee level. ***Significant at the 1% level; **significant at the 5% level; *significant at the 10% level

The findings of a zero WFH effect and a negative hybrid work effect are robust to various changes in the statistical model specification and data preparation. Table C.3 estimates the same regressions as in Table 1, except with a quadratic rather than linear time trend. Table C.4 estimates the same regressions as in Table 1, but drops the top 0.1% of outcomes to investigate the sensitivity of the results to outliers. Table C.5 demonstrates that we also find a significantly negative hybrid effect with binary innovation outcomes (i.e., whether an employee submitted at least one idea) in OLS and logit specifications. We also find a neutral WFH effect, hence the results here are robust when using only the extensive margin of innovation rather than counts as in this section. All these tables can be found in Supplementary Material C.

Finally, since we only observe the date of idea submission in the Idea Portal, but not the date of idea generation, it is possible that ideas have been generated weeks before, so we sometimes attribute them to the wrong work mode. To test the robustness regarding lagged reporting of ideas, we re-estimate the average WFH and hybrid work effects with the outcome variables lagged by one or two months in Table C. 6 in appendix C. The hybrid work effect remains significantly negative in all specifications.

These estimates suggest that it is not so important for innovation whether the workforce works from home or from the office, but it is important that they are consistent. Perhaps counter-intuitively, the hybrid work effect is worse than a convex combination of the WFO and WFH outcomes.

A potential explanation for why hybrid might be worse for innovation than both WFO and WFH is costs of coordination and communication. If everyone is in the office, it is easy to talk to colleagues, and meetings can be spontaneous and in person; “watercooler” conversations can take place. Those conversations can lead to the generation of new ideas, but they can also provide feedback (positive or negative) which might spur refinement or reformulation. If everyone is at home, then similarly all are in the same chat rooms and video meetings, and using the same modes of online communication. It is possible in this case to establish substitute channels of communication. However, under hybrid, some employees are in the office and some are at home, and at varying times throughout the workday. Office employees might talk amongst themselves in person, whereas remote employees talk online. Moreover, scheduling a conversation may be more difficult in hybrid mode, relative to both WFO and WFH. These are additional barriers for the team. Getting everyone to talk is harder due to the different modes of communication. Because of these coordination and communication issues with hybrid work, innovation may suffer.

**Table 2:** OLS: The effect of inequality in office presence on idea quantity.

Dependent variable	(1)	(2)	(3)	(4)
	NumIdeas	NumWeightedIdeas	NumIdeasMS3	NumWeightedIdeasMS3
WFH	0.001	−0.000	0.006	0.000
	(0.001)	(0.001)	(0.004)	(0.002)
Hybrid	−0.005***	−0.004***	−0.021***	−0.015***
	(0.001)	(0.001)	(0.003)	(0.002)
Hybrid $\times$ TeamOfficeDaysSD	−0.046**	−0.025**	−0.159**	−0.072**
	(0.019)	(0.010)	(0.067)	(0.034)
Hybrid $\times$ TeamSize	0.003**	0.001	0.012***	0.005*
	(0.001)	(0.001)	(0.005)	(0.003)
Employee FE	Yes	Yes	Yes	Yes
Team FE	Yes	Yes	Yes	Yes
Month FE	Yes	Yes	Yes	Yes
Linear time trend	Yes	Yes	Yes	Yes
Observations	1039783	1039783	852082	852082
Clusters	47299	47299	43891	43891

NumIdeas and NumWeightedIdeas are the number of submitted ideas and the number of submitted ideas inversely weighted by the number of idea authors, respectively. NumIdeasMS3 and NumWeightedIdeasMS3 are moving 3-month sums of the number of submitted ideas and the number of submitted ideas inversely weighted by the number of idea authors, respectively. TeamOfficeDaysSD is the standard deviation of the days in the office that month among all team members (divided by 100 to rescale). TeamSize is the number of employees in the team (divided by 1000). The unit of observation is the employee-month. Standard errors are shown in brackets below the point estimates, and are clustered on employee level. ***Significant at the 1% level; **significant at the 5% level; *significant at the 10% level

To evaluate this hypothesis, we constructed a measure of the extent to which members of the same team adapt similar hybrid work practices. For each employee, we measured the number of days in the office each month, and then variation in this measure across team members. For example, some teams continued to WFH exclusively, while some were much closer to full WFO, while others were somewhere in between.

Table 2 compares teams with a higher variation in office presence with those that have a lower variation in office presence. Indeed, the former have a significantly worse hybrid effect on the quantity of ideas than employees in teams with low variation in office presence. Hence, teams that are more scattered between office and home innovate less during hybrid (relative to WFO), compared to teams that are less scattered. The interaction effect is significantly negative for all of our four idea quantity measures, which is strong evidence in favor of our conjecture that coordination on a communication channel for informal chats within the team is important. This can explain why teams that are more scattered between office and home are less innovative. That said, the hybrid effect is negative on average even in teams with no variation in office presence; i.e., in teams that either fully WFH or fully WFO. (Among teams with a standard deviation of at most 1 in terms of employee days in the office per team, 89% are fully remote.) Several explanations are possible for the negative effect in well-coordinated teams. One possibility is that substitute communication channels are not being established as rigorously as under WFH, which could be a reason why innovation suffers even if an entire team ends up working from home. But it is also possible that the missing contact to other teams has negative impacts on innovation, in line with the idea that individuals who bridge different teams are often successful innovators^13^.

We conducted several robustness analyses. In Supplement C, Table C. 9 shows that these results are robust to including an interaction between hybrid and the group mean in office attendance, in addition to the group SD as before. Hence, the lower hybrid effect really is due to more variation in office presence, not more (or less) office presence overall. Moreover, Table C. 10 repeats the analysis of Table 2 using the minutes that each employee spent in the office that month, rather than the number of days they were in the office, to compute the office attendance variation measure. The results are very similar.

### Idea quality

While the quantity of ideas is important for innovation, so is the quality of those ideas. A better idea might generate more profit for the firm or more value to the client. Table 3 displays estimates of the WFH and hybrid effect on three measures of idea quality: (i) “IdeaAccepted” indicates whether or not a suggested idea was accepted for implementation; (ii) “ClientShared” indicates whether an idea was shared with a client; (iii) “ClientApproval” indicated whether an idea received a good rating of 3 or 4 (on a 1-4 scale) by the client. As in the quantity regressions, we control for seasonal as well as linear time trends, and we include author-team fixed effects. The sample used includes only ideas where internal review is finished, so these ideas are either accepted or rejected. Hence, informally, the estimates we get are the difference in quality between an idea submitted by the same set of employees during WFH and an idea submitted during WFO, and similarly for hybrid work vs WFO.

**Table 3:** OLS: Average WFH and Hybrid Work Effects on Idea Quality.

Dependent variable	(1)	(2)	(3)
	IdeaAccepted	ClientShared	ClientApproval
WFH	−0.067	−0.090**	−0.183**
	(0.051)	(0.045)	(0.090)
Hybrid	−0.062	−0.001	−0.176
	(0.075)	(0.064)	(0.119)
All ideas finished review	Yes	Yes	Yes
Author-Team FE	Yes	Yes	Yes
Month FE	Yes	Yes	Yes
Linear time trend	Yes	Yes	Yes
Test $\text {WFH}=\text {Hybrid}$ (*p*-value)	0.917	0.117	0.934
WFO Mean	0.867	0.926	0.636
Observations	2898	2656	2898
Clusters	2069	1910	2069

*Note:* IdeaAccepted is an indicator equal to 1 if an idea was accepted for implementation in the internal review, and 0 otherwise. ClientShared is an indicator equal to 1 if an idea was communicated to the client, and 0 otherwise. ClientApproval is an indicator equal to 1 if an idea was rated with 3 or 4 by the client, and 0 otherwise. All regressions use only ideas in a month where more than 50% of submitted ideas were reviewed. The unit of observation is the submitted idea. Standard errors are shown in brackets below the point estimates, and are clustered on author-team level. ***Significant at the 1% level; **significant at the 5% level; *significant at the 10% level

In WFH the quality of submitted ideas is lower than in WFO. In Table 3, for all three quality measures, the sign of the estimated WFH effect is negative. The probability of accepting a suggested idea is 6.7 percentage points lower for ideas submitted during WFH, compared to WFO ideas (Column 1). However, this difference is not statistically significant. The probability of sharing the idea with the client is 9 percentage points lower for ideas submitted during WFH, compared to WFO ideas (Column 2). This difference is both economically and statistically significant. The probability of receiving a high client rating is about 18 percentage points lower for ideas submitted during WFH, compared to WFO ideas. This is a large effect, which is also statistically significantly different from zero.

While the sign of the hybrid work effect is negative for all measures, none of the differences are statistically significant. The regressions in Table 3 use only ideas from months where more than 50% of ideas have been internally reviewed, in order to avoid a potential bias if better ideas are reviewed faster. In a simple regression of the months to a review decision on IdeaAccepted (not displayed), every additional month is estimated to reduce the acceptance probability by 1.6 percentage points ($t=$− 10.34, SEs clustered on author-team level). Since there was less time for review for ideas submitted during hybrid work, this is the work mode that loses observations first as the review rate threshold is increased. At a review rate of above 50%, as in Table 3, only the last 3 months of hybrid drop out of the sample, and WFH as well as WFO retain all months. This means that, if the “fast review selection effect” is not removed due to conditioning on a sufficient review rate, hybrid is favored by the selection effect. Therefore, if anything, the selection goes against WFO and favors hybrid, so the hybrid coefficient in Table 3 may overestimate the real effect.

This discussion raises the question of robustness of results to choice of review rate threshold. Figure C.2 in Supplement C.2 plots the coefficients of the WFH and the Hybrid dummies depending on the review rate threshold, estimated in regressions as in Table 3 but varying the threshold. Since the review rates do not reach 70% for any month during the WFH and hybrid work modes, we cannot estimate these coefficients for a review rate of 70%, hence the upper bound in the figure is 65%. We chose a lower bound of 40% for the review rate, which drops only a single hybrid work month, and so barely corrects for the “fast review selection effect.”

Figure C.2 in Supplement C.2 shows that the WFH coefficient—for outcome IdeaAccepted—is negative for all review rate thresholds and statistically insignificant for all but one review rate threshold (at 60%). The hybrid coefficient is negative and statistically insignificant for all review thresholds. For ClientShared, the WFH coefficient is generally negative (except at the highest review rate), and significantly negative at 45% and 50%. The Hybrid coefficient is generally close to zero and statistically not different from zero for all review rates. For ClientApproval, the WFH coefficient is generally negative, and significantly negative at 45% and 50%. The Hybrid coefficient is generally negative and statistically insignificant.

In summary, the robustness analysis in Figure C.2 demonstrates that there is never a significantly positive WFH or hybrid work effect on idea quality, irrespective of the chosen review rate threshold or quality measure. For almost all specifications, the signs of the effects are negative, the Hybrid effect is always statistically insignificant, and the WFH effect is sometimes significantly negative. Our conclusion is that the Hybrid effect on quality is statistically zero, and the WFH effect on quality is non-positive. That is, for WFH, the evidence is divided between significantly negative and insignificant estimates, with the latter in the majority. As neither a conclusion of no WFH effect nor a conclusion of a negative WFH effect are completely robust, we conclude that WFH has a non-positive effect on idea quality. The zero hybrid effect is remarkable, because idea quantity declines as we have seen above. The evidence in this section shows that it is not the case that the worst ideas are discarded first in this process. Instead, the decrease in innovation seems to affect the good ideas at least as much as the bad ones.

In Supplement C, we get the same results and very similar estimates when using a quadratic rather than linear time trend (Table C. 11). Moreover, we show that these conclusions remain if we assume the idea submission dates were one or two months earlier, to allow for the possibility that ideas were conceived earlier and possibly under a different work mode. These estimates are displayed in Table C. 12. Finally, Supplement Table C. 13 shows heterogeneous effects for the three quality measures.

Did the type of ideas change with work mode? The fraction of process improvement ideas dropped significantly by about 10 percentage points during WFH, relative to WFO, a massive effect (the fraction in the entire sample over all work modes is 26.7%). Both WFH and hybrid significantly increased the fraction of cost reduction ideas compared to WFO, by about 9 percentage points (the hybrid effect is significantly different from zero only at the 10% level). Again, these are large effects; the cost optimization category has a fraction of 19.6% in the entire sample. Last, pure WFH produced more technical solutions than hybrid work.

The change in idea composition does not explain the negative effect of WFH on quality, though. We re-run the regression to estimate the average WFH and hybrid effects in Table 3 (in Supplement C), but include the indicators for the idea categories, to get the average WFH and hybrid effect when holding the idea composition constant. These regressions are displayed in Table C. 14. The qualitative results from before remain: the WFH effect is significantly negative for the client-related idea quality measures, while the hybrid effect remains statistically zero. Thus, the change in idea composition does not explain these negative WFH effects, and in fact, the negative point estimates get slightly more extreme after controlling for the idea composition. Hence, there is something else about WFH that reduces some aspects of idea quality.

## Discussion

Very little is yet known about the effects of working from home or hybrid work on employee innovation. Many corporations express concerns that innovation will suffer. A potential benefit of WFH is that the employee may find it easier to carve out focus time in which they are not interrupted by employee conversations, texts, emails, or meetings. That might help improve the quality of ideas. However, a countervailing consideration is that collaboration is less effective when virtual. One reason could be that communication is more difficult online than in person^9,11^. Since there is some evidence that additional effort improves idea quality more than quantity^10^, the higher communication costs might lead to less effort spent on team innovation, thus lowering quality. Similar arguments apply to hybrid work modes, though probably with milder effect. In principle, an employee might get the best of both worlds with hybrid mode, but for collaboration that will only be true if the employee can arrange to be at the office at the same time as key colleagues. Moreover, both hybrid and home work modes make it significantly less likely, perhaps nearly impossible in the case of WFH, for random interactions that can be one source of innovation.

There is very little evidence yet on these channels, and no previous study with a comparable high-quality measure of innovation. Early studies do suggest reasons to be concerned about innovation with remote work. Virtual communication methods have been found to inhibit generation of creative ideas^8^. In a lab experiment,^14^ find no difference between video conferencing and face-to-face meetings, but that has not been replicated in a real work setting.

Weak network ties are particularly important for innovation^15^. Strong network ties tend to be associated with network homophily (similarity amongst members), density (overlap in relationships between people), and greater similarity of information and ideas. Strong networks also tend to have greater network cohesion, which may stifle new ideas or approaches. By contrast, one’s weak ties provide opportunities to connect with people who are more different in various ways, including information, expertise, and ideas. Extending that idea,^13^ provides evidence that weak ties allow one to bridge “structural holes” across sub-networks, thereby improving a variety of career outcomes, notably including innovation. However, there are tradeoffs in how network structures affect innovation. Closed networks tend to have more efficient communication. Moreover, those with strong ties are more likely to be willing to provide resources to each other. Other research finds that weak ties help a team search for useful knowledge, consistent with Burt’s argument about structural holes^16^. However, they also impede knowledge transfer compared to teams with stronger ties, particularly when new knowledge requires significant translation or communication (rather than being well codified or explicit). Those findings are suggestive of March’s famous distinction between two types of innovation, “exploration” and “exploitation”^17^. Exploration involves searching for new possibilities, while exploitation involves improving existing practices. In our context of IT professionals an example of the former might be a new product or product line extension, while an example of the latter might be a process improvement that increases efficiency or quality of coding.

Previous studies have found decreases in network ties during home working^9,11^. Along similar lines, a study of network contacts at a large North American university found that lack of researcher co-location during the Covid-19 period caused the loss of 4,800 weak ties^18^. When that university shifted to hybrid work, there was partial but not full regeneration of those weak ties. They conclude that employees who do not work in the same location are less likely to form ties and that this weakens the spread of information. In one study software engineers working in two buildings located several blocks apart received 23% more feedback on their computer code if the team was working with all members in the same building^19^. After offices closed during Covid, most of this advantage disappeared. This study also found that sitting near coworkers improved feedback for junior workers and females.

Our findings suggest that an employee’s ability to be innovative, and to collaborate with colleagues, may suffer from working outside of the office. When working from home, the employees in our sample continued to suggest ideas at the same rate, but the quality of those ideas declined. When working in hybrid mode, the quantity of ideas fell relative to fully working in the office. One might have expected that this should lead to an increase in average quality—at least if it is the worse ideas that get dropped first. However, we do not find this to be the case. Idea quality does not change compared to working from the office.

In related work, we found that productivity fell dramatically during WFH compared to WFO^11^. We linked this in part to higher coordination costs. Time spent in meetings and related coordination activities increased. Moreover, employees had less “focus time” (uninterrupted periods of work). We speculate that hybrid may worsen some of these concerns. In hybrid mode, one cannot be sure if a colleague is currently at the office, commuting, or working remotely. This raises costs of connecting with someone, including the time until a meeting (virtual or in-person) can be scheduled. That might shift communication more to emails, or contacting the second-best colleague for an issue, which may be less effective than meeting on Teams or in-person.

These results are important, as they do suggest that the concerns of companies that innovation will suffer with new work modes may be valid. Of course, this cost may be acceptable given the significant benefits for employees in terms of work-life balance^3^. Moreover, companies will gradually learn how to improve innovation with hybrid or WFH work modes. For example, some companies mandate specific weekdays when all employees must be at the office. Many also schedule regular online group meetings via Zoom or Teams. Along similar lines, supervisors might require that employees engaging in close collaboration must be at the office on the same days. Publicly available calendars can make it easier to connect with employees whether working remotely or at the office. Finally, companies might develop better means for employees to meet new colleagues, and to better share information about what different people are working on. Interestingly, there is work suggesting that during hybrid work modes employees endogenously sort into work patterns that increase co-attendance^20^. This suggests that employees may consciously or unconsciously be aware of the benefits of co-attendance.

There are some interesting dimensions of heterogeneity. In particular male employees suffer less of a decline in terms of idea quantity than female employees during hybrid work. One possible reason for this difference is that women simply have less bandwidth to innovate under hybrid or home working modes (e.g. due to other demands placed on them when WFH). Another possible reason could be that women’s work modes differ, particularly under hybrid. We do not find evidence for this. Women go to the office on 0.07 days less per week in the office than men ($p=0.0778$) under the hybrid work scheme and the total time spent in the office is only 10 minutes less per week than that of men on average ($p=0.6929$).

Our analysis is limited in that it is an event study. We are able to control for a variety of important variables, but caution is needed in inferring causality. Rather, our findings are suggestive of the view that innovation and other aspects of work that benefit from employee interactions will suffer under hybrid or full WFH modes, compared to WFO. We hope that our findings will stimulate further research to understand these issues more fully. One interesting avenue for future research could be to study the impact of hybrid and remote work on productive competition between employees, in contrast to collaboration.

A significant benefit of this study is the rare opportunity to analyze high-quality and meaningful measures of innovation in a real workplace setting. Obtaining such data is exceedingly rare. In addition, we are able to study a key question in the current debate about hybrid and remote work modes. Many firms are concerned that various intangible aspects of organizational effectiveness will suffer when employees spend less time physically co-located; e.g., corporate culture, investment in firm-specific human capital, development of professional networks, collaboration, and innovation. A few studies have provided evidence on some of these issues, particularly that communication and network formation are less effective when WFH^21^. Other studies have asked which employees report the greatest work-life balance when workers are assigned randomly to a different number of days in the office within a hybrid scheme^3^. They found that employees with an intermediate number of days in the office report greater work-life balance while at the same not performing worse than their colleagues. Yet very little is known about what happens to innovation across these schemes. We hope that future studies will be able to add to the evidence shown here by studying data from other firms and by conducting randomized experiments on different work schemes.

Last, it is clear that when assessing the benefits of WFH and hybrid work schemes, innovation is only one, albeit an important, aspect. Employee productivity and satisfaction are other important parameters for the firm (see e.g.^11^). These in turn can impact retention and employee innovation in the medium and long term. From a wider economic and social perspective, implications for the environment (due to changing commute patterns) and consequences for urban planning and quality of life are important considerations^21–24^.

### Supplementary Information

Supplementary Information.

## Data Availability

The datasets generated and/or analysed during the current study are not publicly available as they are confidential personnel records, but will be made available to researchers who sign the company’s non-disclosure agreement. Please contact the corresponding author.
