# Supplementary material for: Employee innovation during office work, work from home and hybrid work
Source: Sci Rep. 2024 Jul 24;14:17117. doi: 10.1038/s41598-024-67122-6 (PMC11269655; doi:10.1038/s41598-024-67122-6)
Supplement: Supplementary file 1 — Supplementary Information. [file 41598_2024_67122_MOESM1_ESM.pdf]

Employee Innovation During Office Work, Work from Home and Hybrid Work
# – Online Supplementary Material –

Michael Gibbs[1,2] Friederike Mengel[3,4] Christoph Siemroth[3]

[1]University of Chicago, [2]Institute of Labor Economics, [3]University of Essex, [4]University of Heidelberg

10 June 2024

Contents

A Background **2**

B More Details on Methods **4**

B.1 Data and Variables 4

B.2 Employee/HR variables 5

B.3 Swipe-in data: Office presence during hybrid work 6

C Additional Results **8**

C.1 Additional Tables 8

C.2 Additional Figures 23

A Background

For this study we worked with HCL Technologies, one of the world's largest IT services companies. While it works with clients in many countries, we study employees from a major division who work at its primary corporate campuses in India. The company provides a wide range of technology consulting and outsourcing services for clients, including product and process improvement and R&D to develop new products and services. See [2] and [1] for additional background. Notably, as an IT services company, HCL is in an ideal position to implement WFH or hybrid work, as most employees already used company-provided laptops and mobile phones during the WFO phase. Moreover, the business did well in all three phases, as many clients have sought IT help during and after the pandemic.

Our subjects are highly skilled and educated IT professionals. Virtually all have at least a bachelor's degree, usually in a technology field such as software engineering. Their jobs involve significant cognitive tasks and collaboration with teammates and clients. Importantly, they also involve significant innovation, as they help clients improve processes and products, come up with new products and services, and design and implement those.

While the pandemic presented great challenges in many industries, IT services companies fared well. In this firm, promotion rates were higher in 2020 than in 2019. HCL's total workforce size, revenue, and profit margins rose continuously over our sample period of 2019-2022. Thus employees did not experience any decline in formal or informal incentives during the sample period.

For a more detailed description of the Idea Portal, see [2]. In that paper we analyzed an experiment in which HCL randomly assigned some client teams to be offered a small incentive for submission of ideas that were accepted for implementation. We found that the incentive increased the quality of ideas submitted, and estimated the financial benefits of the incentive to be quite large. As a result of that study, HCL implemented the experimental incentive throughout the company, and it remains in place today.

The Idea Portal data has information on all ideas submitted each month. If an employee is not visible in these data, they did not submit any ideas, as all were eligible and encouraged to participate in the system. In order to evaluate whether the quantity of submitted ideas per employee increased or decreased during WFH and hybrid work, we need to recreate the historical employee rosters, i.e., the sets of employees eligible to submit ideas to the Idea Portal at any given time. This is especially important because the headcount in the division of the firm we study changed over time.

To recreate employee rosters for every month in our sample period, we used complete lists of eligible employees for four different historical dates, April 2019, 2020, 2021, and 2022. For these four points in time, we know exactly which and how many employees were part of the firm and were eligible to submit ideas to the Idea Portal. We interpolate rosters for the months between these discrete points in time as follows.

If an employee is part of a list but not part of the subsequent list, then the employee must have left the firm some time in the 11 months between. We do not know the precise date they left, so we assume they left at the midpoint, i.e., after 6 months (October 31). In Appendix C we confirm that the main results hold under the alternative assumption that employees remained for 11 months,

the longest time consistent with the employee lists. Next, since we know the precise month when each employee joined the firm (see Tenure below), we can accurately add employees to the panel from their first month on the job. If employees joined more than 11 months prior to our first list in April 2019, then we deliberately only extend the panel back at most 11 months. We truncate this way to avoid survivor bias, and choose 11 months to be consistent with the gaps between subsequent lists. Hence, our sample window does not stretch farther back than May 2018, even though there are many employees that joined earlier, because otherwise only very specific (unrepresentative) employees would be part of the early months in the sample.

Thus, we have a list of eligible employees for every month from May 2018 until October 2021, the last month of Idea Portal data.

This study builds on two prior projects in which we analyzed data from this firm. Both used data from the same types of employees in the same part of the firm as studied here. [2] analyzed an experimental incentive tied to suggesting new ideas. The firm uses a formal employee suggestion system, and has found it be an important source of innovation and its ability to increase value-added for its clients. [1] analyzed the effects of the switch from WFO to wFH on employee productivity. We found that productivity fell dramatically, and that a primary cause was an increase in communication costs, and a loss in the employee's ability to have focused work time. The latter effects suggest that innovation might suffer in WFH or HY work modes; that is what is studied here.

B More Details on Methods

B.1 Data and Variables

We use the following measures of idea quantity (per employee per unit of time).

NumIdeas is the number of ideas an employee submitted (possibly with other employees as coauthors) in a given month. If an idea is submitted by more than one employee, then each author's NumIdeas is increased by 1, so there is no weighting by the number of idea authors.

NumWeightedIdeas is the number of ideas, each divided by the number of authors of that idea, that an employee submitted in a given month. Hence, a single author idea increases NumWeightedIdeas by 1, whereas an idea with three authors increases it only by 1/3. This weighting means that, in the aggregate, NumWeightedIdeas gives all ideas the same weight, independent of the number of idea authors.

NumIdeasMS3 is the number of ideas that an employee submitted in a given month and the preceding and subsequent months. Hence, it is a moving sum of ideas over three months. Given that idea submissions are quite infrequent, a potential advantage of the longer aggregation time horizon is that an unusually innovative month does not change the outcomes as much, which might make seasonal time controls in the later regressions more reliable. We define the moving sum only if all of the three months that are aggregated are part of the same work regime. Hence, months directly before and after a regime switch are not used in the analysis, because it would mix the outcomes of different work regimes. As a consequence, the three month moving sum has fewer observations in our panel than the one month aggregated variables above.

Finally, NumWeightedIdeasMS3 is the moving three month sum of NumWeightedIdeas, again only defined if all three months are part of the same work regime. Table B.1 provides summary statistics for these variables by work regime, on the employee-month level.

Table B.1: Summary statistics for idea quantity outcome variables

	Mean	SD	N
**WFO**			
NumIdeas	0.009	0.146	511662
NumWeightedIdeas	0.006	0.099	511662
NumIdeasMS3	0.030	0.271	450179
NumWeightedIdeasMS3	0.018	0.181	450179
**WFH**			
NumIdeas	0.010	0.308	162454
NumWeightedIdeas	0.006	0.142	162454
NumIdeasMS3	0.034	0.688	106772
NumWeightedIdeasMS3	0.019	0.309	106772
**Hybrid**			
NumIdeas	0.007	0.135	395617
NumWeightedIdeas	0.005	0.094	395617
NumIdeasMS3	0.022	0.247	316767
NumWeightedIdeasMS3	0.014	0.170	316767

For our analyses of idea quality, we create a panel of submitted ideas directly from the Idea Portal data, instead of a panel of employees as before, so that the unit of observation is a submitted idea. Our three main outcome variables for idea quality are as follows.

IdeaAccepted is a dummy variable, indicating whether or not a suggested idea was accepted for implementation. The internal review at the company is very rigorous, since implementing implementing bad ideas or not accepting very good ideas has substantial costs to the firm. The company has a panel of senior executive review ideas, which entails significant opportunity cost of their time. Hence, this measure reflects quality from the perspective of the firm very well, as it has a strong profit motive to only accept worthwhile ideas.

ClientShared is a dummy variable, indicating whether an idea was shared with a client. For example, an idea might propose an improvement of a product that the client uses. The firm tried to share ideas with clients from the start of the Idea Portal whenever possible, in order to project an innovative corporate image and to demonstrate value-added to clients. This variable therefore captures not only whether an idea is good, but also whether the idea is suited to make a good impression on outsiders. An idea is shared only if it affects the client directly and if it is good enough.

ClientApproval is a dummy variable, indicating whether an idea received a rating of 3 or 4 by the client. Since there is censoring in the data, the client rating is not useful on its own: Clients only rate an idea if it is a good one, and do not rate it if it is a bad one. Hence, over all ideas in the Portal, only 2.5% of ratings are worse than 3 (on a scale of 1 to 4), which is a one-sided signal with little variation. Improving on this, ClientApproval interprets a good client rating as a good quality signal, and a bad rating or no rating as a bad quality signal. This does not mean that every idea without rating is bad, but it is true that unrated ideas are worse than well rated ideas on average, either from the perspective of the firm (which chose not to share the idea with the client) or the client (who chose not to give a good rating).

In terms of idea properties, we have NumAuthors, which is the number of employees that submit an idea together. This number if capped above at 5 in the submission form. Moreover, when submitting an idea, employees can select an idea category, which classifies it into (in decreasing frequency) process improvement, technical solution, cost optimization, cycle time reduction, tool development, and risk mitigation. We create dummy variables for the largest three categories: IdeaProcess, IdeaTechnical, IdeaCost. These variables give us an idea whether the change in work regime also triggered a change in the kind of ideas that employees come up with. These idea level outcome variables are summarized in Table B.2 by work regime.

B.2 Employee/HR variables

Age is the employee age in years, precise to the month. Hence, an employee who is 20 years and 2 months old is counted as $20 + 2/12$. Experience is the number of years, precise to the month, that the employee has worked in the sector (either in this firm or another). In the raw data we received two separate experience times, 'total experience' that measures the number of years worked and 'relevant experience' that measures the number of years worked in the same sector. Their correlation is .991.

Table B.2: Summary statistics for idea quality outcome variables (only ideas that finished review)

	Mean	SD	N
**WFO**			
IdeaAccepted	0.867	0.340	1645
ClientShared	0.926	0.263	1465
ClientApproval	0.636	0.481	1645
NumAuthors	1.957	1.190	1645
IdeaProcess	0.219	0.414	1645
IdeaTechnical	0.199	0.399	1645
IdeaCost	0.236	0.425	1645
**WFO**			
IdeaAccepted	0.933	0.251	551
ClientShared	0.932	0.251	517
ClientApproval	0.673	0.469	551
NumAuthors	2.005	1.253	551
IdeaProcess	0.169	0.375	551
IdeaTechnical	0.232	0.423	551
IdeaCost	0.303	0.460	551
**Hybrid**			
IdeaAccepted	0.949	0.220	900
ClientShared	0.960	0.195	857
ClientApproval	0.639	0.481	900
NumAuthors	1.742	1.059	900
IdeaProcess	0.218	0.413	900
IdeaTechnical	0.234	0.424	900
IdeaCost	0.252	0.435	900

For this reason, Experience uses relevant experience, in order to avoid collinearity issues in later regressions. Male is a dummy variable indicating a male employee.

The variable NumChildren is the number of children up to age 21 who are covered under the company's employee health insurance plan. Because of its relatively generous health insurance, the company believes that the vast majority of employees who have dependent children insure them via the company. However, some might be insured through a partner's employer. Thus a zero means that there are either no children at home, or there are but they have not been declared.

SalaryBand is the salary group of the employee, from 0 to 7, with a higher number indicating a higher salary group. This is a proxy for hierarchical level. Tenure is the number of years, precise to the month, that the employee has been with the firm. Table C.2 in the appendix shows summary statistics (on employee-month level) for these variables.

B.3 Swipe-in data: Office presence during hybrid work

The campuses at HCL are accessible only with a valid employee ID card. These cards are swept through card readers, which give employees access and record the time of entry to and exit from the office. We obtained these data for the period from October 2020 to December 2021, i.e., the hybrid period we are investigating. These data show, for every day, how many minutes an employee spent at the office.

Based on these data, we compute a measure of how unequal teams are regarding their office presence. To do this, we count the number of days each employee accessed the office in a given month. If an employee is not present in the data in a month (i.e., never swiped their card), then we count it as 0 days if the employee accessed the office in a month before and in a month after. In other words, we fill gaps with zeros. If there is no preceding or following month with a swipe-in, then we count the month as missing for that employee. This is because we do not observe the precise time an employee leaves the company, so no swipe-in might mean the employee left the company rather than worked from home. Once we have these numbers of days in the office for each employee, we take the standard deviation among all members of the team to compute TeamOfficeDaysSD. Thus, if all team members have the same number of days in the office that month—no matter if that number is high or low—then this measure of office presence inequality is zero. Otherwise it is positive, and increases the more scattered team members are across office and home work.

For a robustness analysis, we also compute TeamOfficeMinsSD, which instead uses the sum of minutes in the office, rather than the number of days in the office, for the inequality measure. TeamOfficeDaysMean is the mean number of days in the office among team members that month. TeamSize is the number of team members for which we could compute the number of days in the office that month.

Thus, we can compare the hybrid effect on the number of ideas submitted for teams with homogeneous work mode—where coordination is easier—and for teams that are more scattered between office and home. We do this in Table **??**, by interacting the Hybrid dummy with TeamOfficeDaysSD. We additionally add an interaction between the hybrid dummy and the team size, to make sure the effect of office presence variation we see is not just driven by team size. Moreover, higher variation means different things for different team sizes. In a team of 100, if 10% are in the office, then these 10 can still fruitfully work amongst themselves. In a team of 10, if 10% are in the office, then collaboration is very hard for this single employee. The additional team size control ensures we compare teams of similar sizes. The regression is thus a type of difference-in-difference estimation, where the treatment is the team variation in office attendance. It compares the change in idea quantity for high-variation teams from WFO to WFH/hybrid, with the change for low-variation teams from WFO to WFH/hybrid. The following results can therefore not be explained by other team-specific and time-constant factors that correlate with office presence variation, such as better or worse management, as these are also present during WFO.

C Additional Results

This section contains additional results.

C.1 Additional Tables

We explore which subgroups are most affected by the shifts in the work regimes. Table C.7 interacts the WFH and Hybrid dummy variables with demographic variables, to quantify the effects by demographics. The regressions also control for the demographic variables separately, to estimate the baseline trends during WFO, but these coefficients are not explicitly displayed in the table to save space. As in the table in the main part, we have idea quantity measured in one or three month windows, and with or without number of authors weighting.

We begin with the demographic effects that are significantly different from zero for all four outcome variables. First, the WFH and Tenure interaction is significantly negative for all four outcome variables. This implies that innovation is affected more negatively for employees who have been with the company for longer than for others. This is an interesting result, given that idea quantity if anything increases with Tenure during WFO (see the positive but insignificant Tenure coefficient), and [2] also found that higher tenure employees are more likely to submit ideas. Note that these effects cannot be explained by age or experience in the sector, as these are controlled for separately, and so are purely tenure effects. Moreover, the Hybrid and Tenure interaction is significantly negative as well, similarly indicating that innovation suffers more for more seasoned employees during flexible working.

There are at least two possible explanations for this finding. It could be that employees in slightly more senior or supervisory roles have to pick up some of the decrease in output by others during these regimes and hence have less bandwidth to innovate.[1] It could also be that more senior employees are dis-proportionately affected by coordination costs occurring under WFH and hybrid regimes.

Second, the significantly positive Hybrid and Male interaction term indicates that male employees suffer less of a decline in terms of idea quantity than female employees during hybrid work. In previous work, we found that women's productivity is more negatively affected by working at home [1]. Hence, one possible reason for this difference is that women simply have less bandwidth to innovate under hybrid or home working regimes. Another possible reason could be that women's work regimes differ, particularly under hybrid. We do not find evidence for this. Women go to the office on 0.07 days less per week in the office than men ($p = 0.0778$) under the hybrid work scheme and the total time spent in the office is only 10 minutes less per week than that of men on average ($p = 0.6929$).

Table C.1, column 1, displays estimates of the WFH and hybrid effect on the number of idea authors per submitted ideas. As before, the regression controls for seasonal and linear time trends, but does not include author-team fixed effects, as the number of authors is always constant within author-team. Unlike what Figure **??** suggests, the estimates of both the WFH and hybrid work effect are significantly positive. The estimates of 0.25 to 0.29 imply that there is an additional co-author every fourth or third idea – clearly large effects. The discrepancy with the graphical evidence comes

[1] In [1], we demonstrate that there is indeed some decrease in output during WFO.

from the fact that the regression estimates are relative to the seasonal and linear time trend, and the time trend is estimated to be significantly negative at about –0.02 authors per month. Relative to this long term trend, WFH and hybrid have more idea authors than WFO. However, this result is purely driven by the linear time trend, and the same regression without the linear time trend yields a zero WFH effect and a significantly negative hybrid work effect – the exact opposite. Since the evidence on collaboration, as measured by NumAuthors, is very sensitive to the linear time trend assumption, we view the WFH and hybrid effects as inconclusive.

Table C.1: OLS: Average WFH and Hybrid Work Effects on Collaboration and Idea Category

	(1)	(2)	(3)	(4)
Dependent variable	NumAuthors	IdeaProcess	IdeaTechnical	IdeaCost
WFH	0.250***	-0.099**	0.076	0.092**
	(0.094)	(0.049)	(0.053)	(0.036)
Hybrid	0.291***	-0.042	-0.024	0.093*
	(0.098)	(0.069)	(0.064)	(0.050)
Author-Team FE	No	Yes	Yes	Yes
Month FE	Yes	Yes	Yes	Yes
Linear time trend	Yes	Yes	Yes	Yes
Test WFH = Hybrid (p-value)	0.664	0.232	0.039	0.972
WFO Mean	1.766	0.276	0.207	0.176
Observations	5948	5948	5948	5948
Clusters	3810	3810	3810	3810

Note: NumAuthors is the number of employees that submitted the idea together. IdeaProcess is an indicator equal to 1 if the idea category is , and 0 otherwise. IdeaTechnical is an indicator equal to 1 if the idea category is , and 0 otherwise. IdeaCost is an indicator equal to 1 if the idea category is , and 0 otherwise. The unit of observation is the submitted idea. Standard errors are shown in brackets below the point estimates, and are clustered on author-team level. ***Significant at the 1% level; **significant at the 5% level; *significant at the 10% level.

Table C.1 helps us address a second question. Did the type of ideas change as work regimes changed? For example, with the unexpected introduction of WFH, perhaps some ideas focused on the new remote work mode and internal processes, whereas ideas before may have focused more on the client's products. To test this, we use the idea type (as classified by the submitting employee team). The regressions analyze the fractions of the three biggest idea categories. We merged the remaining smallest three categories and found no significant WFH or hybrid effects in this "other" category.

Table C.2 displays the summary statistics for the demographic variables that we use to explore heterogeneous WFH and Hybrid work effects. The data is on employee-month level, so each employee can be present in multiple months and in different work regimes.

Table C.3 estimates the average WFH and hybrid work effect as in the main paper, but using a quadratic time trend rather than a linear time trend. The point estimates of the WFH and hybrid work effects are very similar, and all qualitative results are the same. In particular, we again find a zero effect of WFH on idea quantity, and a statistically significant negative effect of hybrid work.

Table C.2: Summary statistics for demographic variables (employee-month level)

	Mean	SD	N
**WFO**			
Age	31.333	5.961	511662
Male	0.765	0.424	511662
Tenure	3.546	4.065	511662
Experience	7.538	5.325	510870
SalaryBand	1.615	0.888	511662
NumChildren	0.471	0.712	511662
**WFH**			
Age	31.850	6.184	162454
Male	0.746	0.435	162454
Tenure	3.908	4.163	162454
Experience	7.928	5.514	162154
SalaryBand	1.641	0.905	162454
NumChildren	0.486	0.721	162454
**Hybrid**			
Age	31.766	6.396	395617
Male	0.732	0.443	395617
Tenure	3.754	4.216	395617
Experience	7.465	5.875	389880
SalaryBand	1.624	0.883	395617
NumChildren	0.463	0.712	395617

Table C.4 estimates the average WFH and hybrid work effect as in the main part of the paper, but dropping the top 0.1% of outcomes.[2] This allows us to see if a few outliers are responsible for the difference between hybrid work and WFO. The regressions show this is not the case: all the significant differences remain statistically significant at high confidence levels even after dropping potential outliers.

Table C.5 estimates the average WFH and hybrid work effect on whether a given employee in a given month submitted at least one idea (which we call 'ideation'). That is, unlike in the main section where we explain the count of submitted ideas, we drop the intensive margin here and just explain the extensive margin for robustness.

Column 1 of Table C.5 estimates a linear probability model via OLS with employee and team fixed effects and time controls. As in the main section with counts, the regression shows a significantly negative effect during hybrid work on innovation activity, relative to working from the office. Work from home, on the other hand, has no significant difference in terms of the probability of generating ideas compared to work from the office. In column 2, the same model is estimated via conditional (fixed effects) logit,[3] which makes only use of observations of employees who switched from ideating to not ideating, hence the number of observations is much smaller. But the results are exactly the same, hence the results from the main section are robust to using only a binary innovation outcome

[2] 0.1% of the top outcomes is substantial, given that NumIdeas is zero for slightly more than 99% observations – innovation on a one-month time scale is quite infrequent.

[3] Note that we were unable to estimate team fixed effects in the logit model, as the number of parameters to be estimated was too high. However, based on the OLS regressions, we know that including the team fixed effects does not change the estimates much, since employees do not change teams very often.

Table C.3: OLS: Average WFH and Hybrid Work Effects (Quadratic Time Trend)

	(1)	(2)	(3)	(4)
Dependent variable	NumIdeas	NumWeightedIdeas	NumIdeasMS3	NumWeightedIdeasMS3
WFH	0.001	-0.000	0.004	-0.000
	(0.001)	(0.000)	(0.004)	(0.002)
Hybrid	-0.005***	-0.004***	-0.026***	-0.017***
	(0.001)	(0.001)	(0.004)	(0.002)
Employee FE	Yes	Yes	Yes	Yes
Team FE	Yes	Yes	Yes	Yes
Month FE	Yes	Yes	Yes	Yes
Quadratic time trend	Yes	Yes	Yes	Yes
Test WFH = Hybrid (p-value)	0.000	0.000	0.000	0.000
WFO Mean	0.009	0.006	0.030	0.018
Observations	1060648	1060648	867234	867234
Clusters	48110	48110	44453	44453

Note: NumIdeas and NumWeightedIdeas are the number of submitted ideas and the number of submitted ideas inversely weighted by the number of idea authors, respectively. NumIdeasMS3 and NumWeightedIdeasMS3 are moving 3-month sums of the number of submitted ideas and the number of submitted ideas inversely weighted by the number of idea authors, respectively. The unit of observation is the employee-month. Standard errors are shown in brackets below the point estimates, and are clustered on employee level. ***Significant at the 1% level; **significant at the 5% level; *significant at the 10% level.

and robust to using linear or nonlinear binary regression models.

In the Value Portal data, we observe the exact day and time when an idea is submitted to the system. However, it is conceivable that some ideas are actually generated weeks before. This might mean that an idea is incorrectly attributed to a later work regime rather than the current one. To test the robustness of our results to the lagged reporting of ideas, we compute the number of ideas and weighted ideas as before, but with a lag of one and two months. The estimates are displayed in Table C.6. The average effect of hybrid work, relative to WFO, is significantly negative in all columns (i.e., for all lags), so the main result is very robust. Interestingly, with a longer lag, the WFH effect becomes significantly positive, unlike in the main section, but only if ideas are not weighted by the number of idea authors.

Table C.8 estimates the average WFH and Hybrid effect separately for the number of single authored ideas and the number of multi authored ideas. This tells us whether the negative average hybrid effects we see in the main section are driven mostly by collaboration (in which case multi author ideas would drop more) or something else (then also the number of single authored ideas would drop).

Table C.9 is similar to Table **??** in the main part, except it adds an interaction between the hybrid dummy and the mean of the days in the office that month in the team (Hybrid × TeamOfficeDaysMean). The result that the hybrid effect is lower for teams with larger inequality in office presence, compared to teams with smaller inequality, remains for all four idea quantity measures.

Table C.10 is similar to the table in the main part, except it uses the minutes spend in the office

Table C.4: OLS: Average WFH and Hybrid Work Effects (top 0.1% of outcomes truncated)

	(1)	(2)	(3)	(4)
Dependent variable	NumIdeas	NumWeightedIdeas	NumIdeasMS3	NumWeightedIdeasMS3
WFH	-0.000	-0.000	-0.001	-0.000
	(0.000)	(0.000)	(0.001)	(0.001)
Hybrid	-0.002***	-0.001***	-0.011***	-0.007***
	(0.000)	(0.000)	(0.002)	(0.001)
Employee FE	Yes	Yes	Yes	Yes
Team FE	Yes	Yes	Yes	Yes
Month FE	Yes	Yes	Yes	Yes
Linear time trend	Yes	Yes	Yes	Yes
Observations	1059231	1057724	865629	865560
Clusters	48110	48110	44453	44453

Note: NumIdeas and NumWeightedIdeas are the number of submitted ideas and the number of submitted ideas inversely weighted by the number of idea authors, respectively. NumIdeasMS3 and NumWeightedIdeasMS3 are moving 3-month sums of the number of submitted ideas and the number of submitted ideas inversely weighted by the number of idea authors, respectively. The unit of observation is the employee-month. Standard errors are shown in brackets below the point estimates, and are clustered on employee level. The top 0.1% of outcomes are discarded before running the regression to deal with potential outliers. ***Significant at the 1% level; **significant at the 5% level; *significant at the 10% level.

that month, rather than the number of days present in the office, to compute team inequality in office presence. The results are very similar, with a significantly negative interaction of the hybrid dummy and the office presence inequality measure. But for the last quantity measure, the interaction is significantly different from zero only at the 10% level. All of these robustness analyses strongly suggest the finding (that office presence inequality has a negative effect on innovation during hybrid) is robust.

Table C.11 estimates the quality regressions from the main section, but uses a quadratic rather than a linear time trend. The qualitative results are exactly the same as the table in the main part of the paper: a non-positive WFH effect, and statistically a zero hybrid effect.

Table C.12 estimates the quality regressions from the main section, but lags the idea submission dates by 1 or 2 months, in order to test the robustness regarding delayed submission of ideas that were conceived earlier (and possibly under a different work regime). The table shows that all signs remain negative for all quality measures and for both the WFH and the hybrid work effect. The significantly negative WFH effect on ClientApproval remains significantly negative, with the magnitude of the effect slightly increasing. The significantly negative WFH effect on ClientShared is now only significantly different from zero at the 10% level. However, all of this is consistent with the main section, which concluded a non-positive effect of WFH and a zero effect for hybrid work on idea quality.

Table C.5: OLS: Average WFH and Hybrid Work Effects on ideation activity

	(1) OLS	(2) Conditional Logit
Dependent variable	Ideated	Ideated
WFH	-0.000	0.015
	(0.000)	(0.056)
Hybrid	-0.003***	-0.479***
	(0.000)	(0.069)
Employee FE	Yes	Yes
Team FE	Yes	No
Month FE	Yes	Yes
Linear time trend	Yes	Yes
Observations	1060648	143260
Clusters	48110	4180

Note: Ideated is 1 if and only if the employee submitted at least one idea in the given month, and 0 otherwise. The unit of observation is the employee-month. Standard errors are shown in brackets below the point estimates, and are clustered on employee level. ***Significant at the 1% level; **significant at the 5% level; *significant at the 10% level.

Table C.6: OLS: Average WFH and Hybrid Work Effects with Lags

	(1)	(2)	(3)	(4)
Dependent variable	NumIdeasLag1	NumWeightedIdeasLag1	NumIdeasLag2	NumWeightedIdeasLag2
WFH	0.002*	0.000	0.002**	0.000
	(0.001)	(0.001)	(0.001)	(0.001)
Hybrid	-0.006***	-0.004***	-0.002**	-0.002***
	(0.001)	(0.001)	(0.001)	(0.001)
Employee FE	Yes	Yes	Yes	Yes
Team FE	Yes	Yes	Yes	Yes
Month FE	Yes	Yes	Yes	Yes
Linear time trend	Yes	Yes	Yes	Yes
Test WFH = Hybrid (p-value)	0.000	0.000	0.000	0.001
WFO Mean	0.010	0.006	0.010	0.006
Observations	1060648	1060648	1060648	1060648
Clusters	48110	48110	48110	48110

Note: NumIdeasLagX and NumWeightedIdeasLagX are the number of submitted ideas and the number of submitted ideas inversely weighted by the number of idea authors, respectively, X months prior to the current month. The unit of observation is the employee-month. Standard errors are shown in brackets below the point estimates, and are clustered on employee level. ***Significant at the 1% level; **significant at the 5% level; *significant at the 10% level.

Table C.7: OLS: WFH and Hybrid Work Effects by Demographics

	(1)	(2)	(3)	(4)
Dependent variable	NumIdeas	NumWeightedIdeas	NumIdeasMS3	NumWeightedIdeasMS3
WFH	-0.000	-0.005	0.009	-0.018
	(0.009)	(0.004)	(0.038)	(0.015)
WFH × Age	0.000	0.000	-0.000	0.001
	(0.000)	(0.000)	(0.002)	(0.001)
WFH × Male	0.003**	0.001	0.011**	0.004
	(0.001)	(0.001)	(0.005)	(0.003)
WFH × Tenure	-0.001**	-0.000**	-0.003**	-0.001*
	(0.000)	(0.000)	(0.001)	(0.001)
WFH × Experience	0.000	-0.000	0.001	-0.000
	(0.000)	(0.000)	(0.002)	(0.001)
WFH × SalaryBand	-0.001	0.000	-0.007	-0.002
	(0.001)	(0.001)	(0.005)	(0.003)
WFH × NumChildren	0.003*	0.001	0.015*	0.005
	(0.002)	(0.001)	(0.008)	(0.003)
Hybrid	-0.008**	-0.005**	-0.030**	-0.017**
	(0.004)	(0.002)	(0.013)	(0.008)
Hybrid × Age	0.000	-0.000	0.000	-0.000
	(0.000)	(0.000)	(0.001)	(0.000)
Hybrid × Male	0.002*	0.001**	0.006**	0.005***
	(0.001)	(0.001)	(0.003)	(0.002)
Hybrid × Tenure	-0.001***	-0.000**	-0.002**	-0.001*
	(0.000)	(0.000)	(0.001)	(0.000)
Hybrid × Experience	0.000	0.000	0.001	0.001
	(0.000)	(0.000)	(0.001)	(0.001)
Hybrid × SalaryBand	0.000	0.000	-0.001	0.000
	(0.001)	(0.001)	(0.003)	(0.002)
Hybrid × NumChildren	0.001	0.000	0.001	0.001
	(0.001)	(0.001)	(0.003)	(0.002)
Demographic controls	Yes	Yes	Yes	Yes
Employee FE	Yes	Yes	Yes	Yes
Team FE	Yes	Yes	Yes	Yes
Month FE	Yes	Yes	Yes	Yes
Linear time trend	Yes	Yes	Yes	Yes
Observations	1054072	1054072	862363	862363
Clusters	47389	47389	43732	43732

Note: NumIdeas and NumWeightedIdeas are the number of submitted ideas and the number of submitted ideas inversely weighted by the number of idea authors, respectively. NumIdeasMS3 and NumWeightedIdeasMS3 are moving 3-month sums of the number of submitted ideas and the number of submitted ideas inversely weighted by the number of idea authors, respectively. The unit of observation is the employee-month. Standard errors are shown in brackets below the point estimates, and are clustered on employee level. ***Significant at the 1% level; **significant at the 5% level; *significant at the 10% level.

Table C.8: OLS: Average WFH and Hybrid Work Effects: Single vs Multi Author Ideas

	(1)	(2)	(3)
Dependent variable	NumSingleAuthorIdeas	NumMultiAuthorIdeas	NumWeightedMultiAuthorIdeas
WFH	-0.000	0.001**	0.000
	(0.000)	(0.001)	(0.000)
Hybrid	-0.003***	-0.003***	-0.001***
	(0.001)	(0.001)	(0.000)
Employee FE	Yes	Yes	Yes
Team FE	Yes	Yes	Yes
Month FE	Yes	Yes	Yes
Linear time trend	Yes	Yes	Yes
Test WFH = Hybrid (p-value)	0.000	0.000	0.000
WFO Mean	0.003	0.006	0.002
Observations	1060648	1060648	1060648
Clusters	48110	48110	48110

Note: NumSingleAuthorIdeas is the number of submitted ideas with a single author. NumMultiAuthorIdeas and NumWeightedMultiAuthorIdeas are the numbers of submitted ideas with more than one coauthor, without and with weighting by the number of idea authors, respectively. The unit of observation is the employee-month. Standard errors are shown in brackets below the point estimates, and are clustered on employee level. ***Significant at the 1% level; **significant at the 5% level; *significant at the 10% level.

Table C.9: OLS: The effect of office presence inequality on idea quantity, controlling for mean office presence

	(1)	(2)	(3)	(4)
Dependent variable	NumIdeas	NumWeightedIdeas	NumIdeasMS3	NumWeightedIdeasMS3
WFH	0.001	-0.000	0.005	-0.001
	(0.001)	(0.001)	(0.004)	(0.002)
Hybrid	-0.005***	-0.004***	-0.021***	-0.015***
	(0.001)	(0.001)	(0.003)	(0.002)
Hybrid × TeamOfficeDaysSD	-0.105***	-0.052**	-0.426***	-0.205***
	(0.035)	(0.021)	(0.120)	(0.069)
Hybrid × TeamOfficeDaysMean	0.066*	0.030	0.301***	0.149**
	(0.036)	(0.023)	(0.107)	(0.071)
Hybrid × TeamSize	0.003**	0.001	0.013***	0.005*
	(0.001)	(0.001)	(0.005)	(0.003)
Employee FE	Yes	Yes	Yes	Yes
Team FE	Yes	Yes	Yes	Yes
Month FE	Yes	Yes	Yes	Yes
Linear time trend	Yes	Yes	Yes	Yes
Observations	1039783	1039783	852082	852082
Clusters	47299	47299	43891	43891

Note: NumIdeas and NumWeightedIdeas are the number of submitted ideas and the number of submitted ideas inversely weighted by the number of idea authors, respectively. NumIdeasMS3 and NumWeightedIdeasMS3 are moving 3-month sums of the number of submitted ideas and the number of submitted ideas inversely weighted by the number of idea authors, respectively. TeamOfficeDaysSD and TeamOfficeDaysMean are the standard deviation and mean, respectively, of the days in the office that month among all team members (divided by 100 to rescale). TeamSize is the number of employees in the team (divided by 1000). The unit of observation is the employee-month. Standard errors are shown in brackets below the point estimates, and are clustered on employee level. ***Significant at the 1% level; **significant at the 5% level; *significant at the 10% level.

Table C.10: OLS: The effect of office presence inequality on idea quantity during hybrid, with minutes rather than days in the office

	(1)	(2)	(3)	(4)
Dependent variable	NumIdeas	NumWeightedIdeas	NumIdeasMS3	NumWeightedIdeasMS3
WFH	0.001	-0.000	0.006	0.000
	(0.001)	(0.001)	(0.004)	(0.002)
Hybrid	-0.005***	-0.004***	-0.022***	-0.016***
	(0.001)	(0.001)	(0.003)	(0.002)
Hybrid × TeamOfficeMinsSD	-0.006**	-0.004**	-0.022**	-0.011*
	(0.003)	(0.002)	(0.011)	(0.006)
Hybrid × TeamSize	0.002**	0.001	0.012***	0.004*
	(0.001)	(0.001)	(0.004)	(0.003)
Employee FE	Yes	Yes	Yes	Yes
Team FE	Yes	Yes	Yes	Yes
Month FE	Yes	Yes	Yes	Yes
Linear time trend	Yes	Yes	Yes	Yes
Observations	1039783	1039783	852082	852082
Clusters	47299	47299	43891	43891

Note: NumIdeas and NumWeightedIdeas are the number of submitted ideas and the number of submitted ideas inversely weighted by the number of idea authors, respectively. NumIdeasMS3 and NumWeightedIdeasMS3 are moving 3-month sums of the number of submitted ideas and the number of submitted ideas inversely weighted by the number of idea authors, respectively. TeamOfficeMinsSD is the standard deviation of the minutes in the office that month among all team members (divided by 10,000 to rescale). TeamSize is the number of employees in the team (divided by 1000). The unit of observation is the employee-month. Standard errors are shown in brackets below the point estimates, and are clustered on employee level. ***Significant at the 1% level; **significant at the 5% level; *significant at the 10% level.

Table C.11: OLS: Average WFH and Hybrid Work Effects on Idea Quality (Quadratic Time Trend)

	(1)	(2)	(3)
Dependent variable	IdeaAccepted	ClientShared	ClientApproval
WFH	-0.053	-0.102**	-0.195**
	(0.053)	(0.052)	(0.097)
Hybrid	0.003	-0.059	-0.228
	(0.099)	(0.094)	(0.170)
All ideas finished review	Yes	Yes	Yes
Author-Team FE	Yes	Yes	Yes
Month FE	Yes	Yes	Yes
Quadratic time trend	Yes	Yes	Yes
Test WFH = Hybrid (p-value)	0.438	0.521	0.788
WFO Mean	0.867	0.926	0.636
Observations	2898	2656	2898
Clusters	2069	1910	2069

Note: IdeaAccepted is an indicator equal to 1 if an idea was accepted for implementation in the internal review, and 0 otherwise. ClientShared is an indicator equal to 1 if an idea was communicated to the client, and 0 otherwise. ClientApproval is an indicator equal to 1 if an idea was rated with 3 or 4 by the client, and 0 otherwise. All regressions use only ideas in a month where more than 50% of submitted ideas were reviewed. The unit of observation is the submitted idea. Standard errors are shown in brackets below the point estimates, and are clustered on author-team level. ***Significant at the 1% level; **significant at the 5% level; *significant at the 10% level.

Table C.12: OLS: Average WFH and Hybrid Work Effects on Idea Quality

	(1)	(2)	(3)	(4)	(5)	(6)
Dependent variable	IdeaAcceptedLag1	ClientSharedLag1	ClientApprovalLag1	IdeaAcceptedLag2	ClientSharedLag2	ClientApprovalLag2
WFH	-0.049	-0.085*	-0.222***	-0.036	-0.075*	-0.210**
	(0.045)	(0.044)	(0.084)	(0.047)	(0.041)	(0.086)
Hybrid	-0.048	-0.052	-0.241**	-0.033	-0.044	-0.173
	(0.059)	(0.057)	(0.112)	(0.062)	(0.058)	(0.112)
All ideas finished review	Yes	Yes	Yes	Yes	Yes	Yes
Author-Team FE	Yes	Yes	Yes	Yes	Yes	Yes
Month FE	Yes	Yes	Yes	Yes	Yes	Yes
Linear time trend	Yes	Yes	Yes	Yes	Yes	Yes
Test WFH = Hybrid (p-value)	0.990	0.537	0.831	0.926	0.540	0.681
WFO Mean	0.871	0.871	0.871	0.871	0.871	0.871
Observations	2986	2740	2986	2988	2741	2988
Clusters	2121	1959	2121	2120	1958	2120

Note: IdeaAcceptedLagX is an indicator equal to 1 if an idea was accepted for implementation in the internal review, and 0 otherwise, where the date of idea submission is reduced by X months. ClientSharedLagX is an indicator equal to 1 if an idea was communicated to the client, and 0 otherwise, where the date of idea submission is reduced by X months. ClientApprovalLagX is an indicator equal to 1 if an idea was rated with 3 or 4 by the client, and 0 otherwise, where the date of idea submission is reduced by X months. All regressions use only ideas in a month where more than 50% of submitted ideas were reviewed. The unit of observation is the submitted idea. Standard errors are shown in brackets below the point estimates, and are clustered on author-team level. ***Significant at the 1% level; **significant at the 5% level; *significant at the 10% level.

In Table C.13, we split the WFH and hybrid work effects by demographics. Since an observation is an idea, which can be submitted by multiple employees, we aggregate the individual demographic variables by taking the mean for the author team. For example, for a team of two employees, AvAge takes the mean of the two Age values, and similarly AvMale is the mean Male dummy of all contributing employees, etc. None of the demographics significantly change the WFH or hybrid effect for all idea quality outcomes.

Interestingly, more experienced teams have a smaller hybrid effect on the probability of idea acceptance than less experienced teams. An additional average year of experience reduces the hybrid effect by about 5.3 percentage points. But for another idea quality outcome—ClientShared—the effect is flipped: One more year of average experience increases the hybrid effect on the probability of sharing the idea with the client by about 3.3 percentage points. Hence, experience does not have a consistent effect.

Older teams have a smaller hybrid effect on the probability of sharing the idea with the client than younger teams. An additional average year of age decreases the hybrid effect by about 3.0 percentage points.

Table C.13: OLS: WFH and Hybrid Work Effects on Idea Quality by Demographics

	(1)	(2)	(3)
Dependent variable	IdeaAccepted	ClientShared	ClientApproval
WFH	-0.179	0.271	-0.666
	(0.247)	(0.361)	(0.633)
WFH × AvAge	0.003	-0.008	0.015
	(0.009)	(0.014)	(0.024)
WFH × AvMale	-0.017	-0.103	-0.079
	(0.082)	(0.089)	(0.159)
WFH × AvTenure	0.018**	-0.011	-0.000
	(0.007)	(0.011)	(0.019)
WFH × AvExp	-0.018	-0.003	-0.030
	(0.014)	(0.017)	(0.035)
WFH × AvSalary	0.032	0.040	0.181
	(0.052)	(0.064)	(0.124)
WFH × AvChildren	0.071	0.041	-0.065
	(0.052)	(0.066)	(0.134)
Hybrid	-0.480	0.883**	-0.236
	(0.392)	(0.403)	(0.922)
Hybrid × AvAge	0.023	-0.039**	-0.009
	(0.016)	(0.015)	(0.037)
Hybrid × AvMale	-0.037	-0.008	-0.009
	(0.078)	(0.120)	(0.202)
Hybrid × AvTenure	0.015**	-0.000	0.012
	(0.007)	(0.008)	(0.021)
Hybrid × AvExp	-0.053**	0.033*	-0.003
	(0.023)	(0.018)	(0.050)
Hybrid × AvSalary	0.078	0.001	0.104
	(0.057)	(0.074)	(0.125)
Hybrid × AvChildren	0.030	0.109**	0.057
	(0.044)	(0.054)	(0.107)
Demographic controls	Yes	Yes	Yes
All ideas finished review	Yes	Yes	Yes
Author-Team FE	Yes	Yes	Yes
Month FE	Yes	Yes	Yes
Linear time trend	Yes	Yes	Yes
Observations	2892	2650	2892
Clusters	2067	1908	2067

Note: IdeaAccepted is an indicator equal to 1 if an idea was accepted for implementation in the internal review, and 0 otherwise. ClientShared is an indicator equal to 1 if an idea was communicated to the client, and 0 otherwise. ClientApproval is an indicator equal to 1 if an idea was rated with 3 or 4 by the client, and 0 otherwise. All regressions use only ideas in a month where more than 50% of submitted ideas were reviewed. The unit of observation is the submitted idea. Standard errors are shown in brackets below the point estimates, and are clustered on author-team level. ***Significant at the 1% level; **significant at the 5% level; *significant at the 10% level.

Table C.14: OLS: Average WFH and Hybrid Work Effects on Idea Quality, Holding Idea Type Composition Constant

	(1)	(2)	(3)
Dependent variable	IdeaAccepted	ClientShared	ClientApproval
WFH	-0.073	-0.096**	-0.192**
	(0.052)	(0.045)	(0.090)
Hybrid	-0.066	-0.003	-0.183
	(0.074)	(0.064)	(0.120)
IdeaProcess	0.011	-0.016	-0.082*
	(0.031)	(0.031)	(0.043)
IdeaTechnical	0.027	0.029	-0.016
	(0.026)	(0.024)	(0.045)
IdeaCost	0.066**	0.024	0.033
	(0.031)	(0.033)	(0.048)
All ideas finished review	Yes	Yes	Yes
Author-Team FE	Yes	Yes	Yes
Month FE	Yes	Yes	Yes
Linear time trend	Yes	Yes	Yes
Test WFH = Hybrid (p-value)	0.898	0.103	0.917
WFO Mean	0.867	0.926	0.636
Observations	2898	2656	2898
Clusters	2069	1910	2069

Note: IdeaAccepted is an indicator equal to 1 if an idea was accepted for implementation in the internal review, and 0 otherwise. ClientShared is an indicator equal to 1 if an idea was communicated to the client, and 0 otherwise. ClientApproval is an indicator equal to 1 if an idea was rated with 3 or 4 by the client, and 0 otherwise. All regressions use only ideas in a month where more than 50% of submitted ideas were reviewed. The unit of observation is the submitted idea. Standard errors are shown in brackets below the point estimates, and are clustered on author-team level. ***Significant at the 1% level; **significant at the 5% level; *significant at the 10% level.

C.2 Additional Figures

Appendix Figure C.1 plots our three idea quality measures. First, a drop in the very last month is noticeable for all quality measures. The last month represents the most recent ideas in the sample, and many of these have not been reviewed yet. Hence, this month's reviewed ideas are a systematic selection, which we will control for in the regressions by requiring that a sufficient share of ideas that month has to have been reviewed. Second, there is a lot of variation month by month for all measures, which makes it hard to gauge WFH or hybrid work effects visually. Third, especially for IdeaAccepted, there is a general long term time trend which is independent of the work regime and which we will also have to control for in order to get a reliable estimate of the WFH and hybrid work effects.

References

[1] M. Gibbs, F. Mengel, and C. Siemroth. Work from home and productivity: Evidence from personnel and analytics data on information technology professionals. *Journal of Political Economy – Microeconomics*, 1(1):7–41, 2023.

[2] M. Gibbs, S. Neckermann, and C. Siemroth. A field experiment in motivating employee ideas. *Review of Economics and Statistics*, 99(4):577–590, 2017.

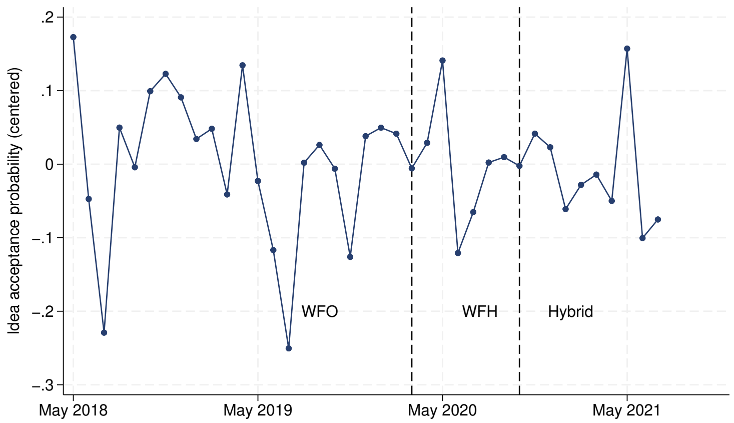

(a) IdeaAccepted

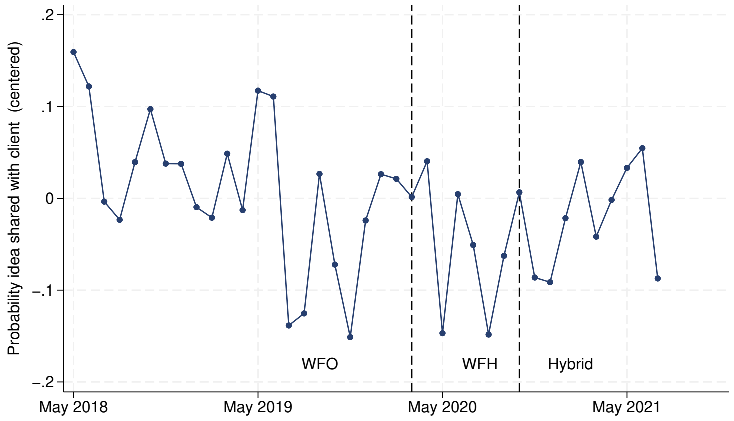

(b) ClientShared

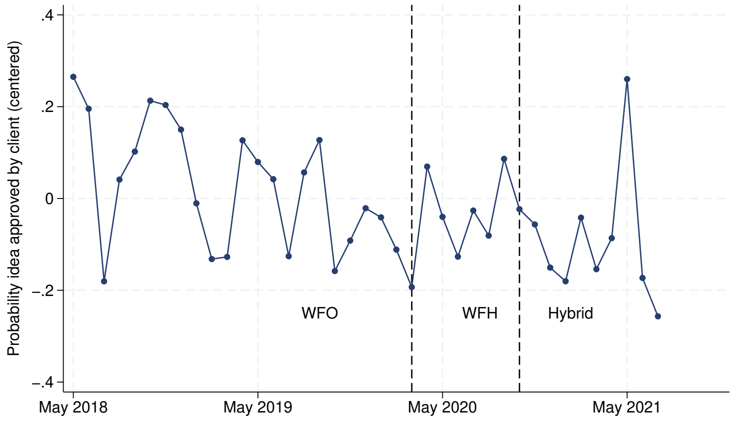

(c) ClientApproval

Figure C.1: The graphs plot each of our three idea quality measures over time after removing the linear and seasonal time trends (y-axis normalized: 0 is WFO mean). The vertical bars indicate changes in the work regime (WFO, WFH, hybrid).

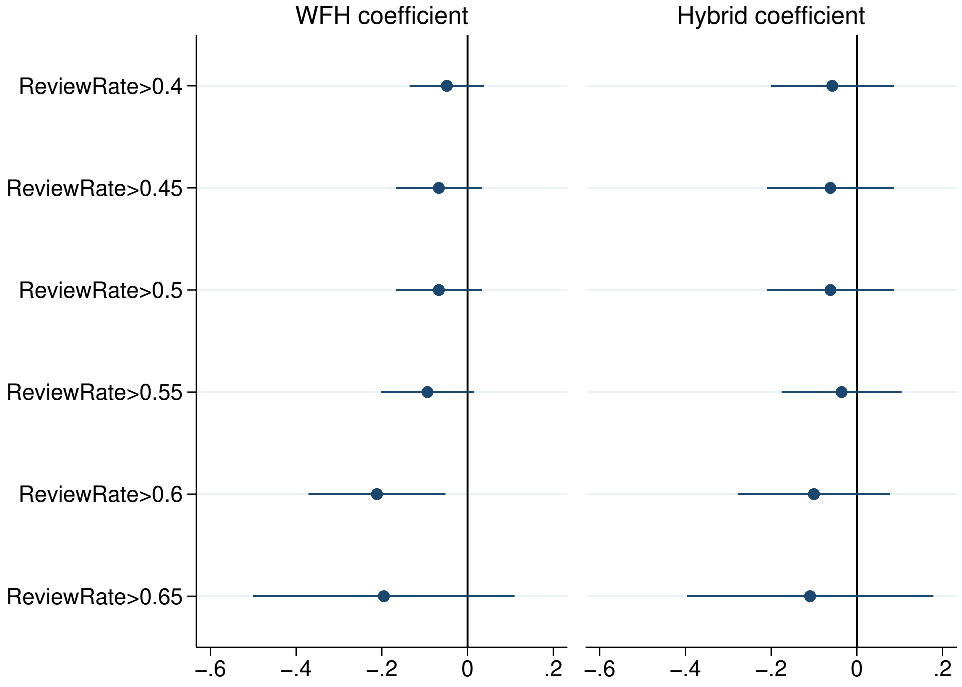

(a) IdeaAccepted

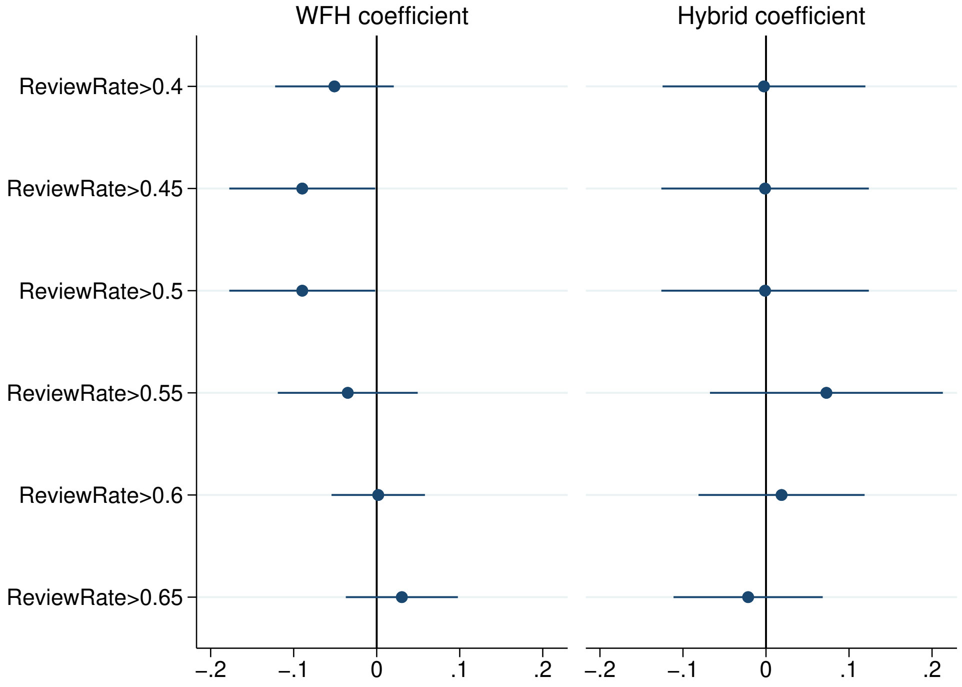

(b) ClientShared

Figure C.2: The regression coefficients of the WFH and Hybrid effects, where the sample depends on the review rate. That is, regression estimation uses only ideas from months where a larger share of x ideas have been reviewed. The graphs plot the coefficients depending on x. (continued next page)

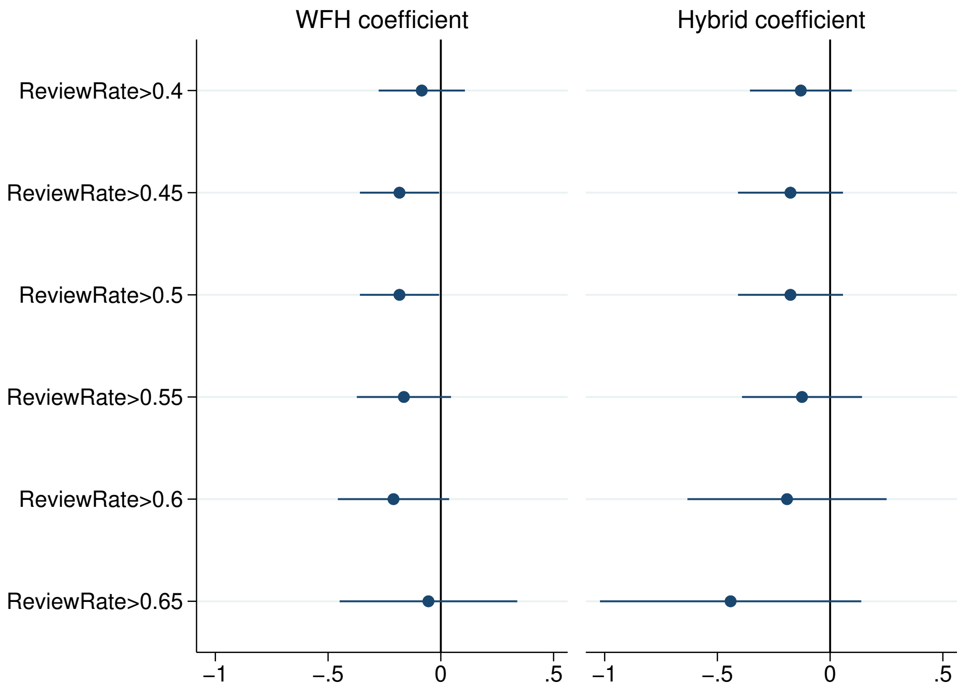

(a) ClientApproval

Figure C.2: (continued) The regression coefficients of the WFH and Hybrid effects, where the sample depends on the review rate. That is, regression estimation uses only ideas from months where a larger share of x ideas have been reviewed. The graphs plot the coefficients depending on x.
